# Recent Developments in Lead and Lead-Free Halide Perovskite Nanostructures towards Photocatalytic CO_2_ Reduction

**DOI:** 10.3390/nano10122569

**Published:** 2020-12-21

**Authors:** Chaitanya B. Hiragond, Niket S. Powar, Su-Il In

**Affiliations:** Department of Energy Science & Engineering, DGIST, 333 Techno Jungang-daero, Hyeonpung-eup, Dalseong-gun, Daegu 42988, Korea; chetan123@dgist.ac.kr (C.B.H.); niketpowar@dgist.ac.kr (N.S.P.)

**Keywords:** lead halide perovskites, lead-free perovskites, CO_2_ reduction, photocatalysis

## Abstract

Perovskite materials have been widely considered as emerging photocatalysts for CO_2_ reduction due to their extraordinary physicochemical and optical properties. Perovskites offer a wide range of benefits compared to conventional semiconductors, including tunable bandgap, high surface energy, high charge carrier lifetime, and flexible crystal structure, making them ideal for high-performance photocatalytic CO_2_ reduction. Notably, defect-induced perovskites, for example, crystallographic defects in perovskites, have given excellent opportunities to tune perovskites’ catalytic properties. Recently, lead (Pb) halide perovskite and their composites or heterojunction with other semiconductors, metal nanoparticles (NPs), metal complexes, graphene, and metal-organic frameworks (MOFs) have been well established for CO_2_ conversion. Besides, various halide perovskites have come under focus to avoid the toxicity of lead-based materials. Therefore, we reviewed the recent progress made by Pb and Pb-free halide perovskites in photo-assisted CO_2_ reduction into useful chemicals. We also discussed the importance of various factors like change in solvent, structure defects, and compositions in the fabrication of halide perovskites to efficiently convert CO_2_ into value-added products.

## 1. Introduction

Photocatalytic CO_2_ reduction, which transforms solar energy into usable chemical fuels, has drawn considerable attention for solving environmental pollution and global energy problems [1,2,3,4,5,6,7,8,9,10,11]. Many attempts have been taken to upgrade the photocatalytic CO_2_ conversion process in terms of material design. In any event, the discovery of a new photocatalyst for better synergistic performance has never stopped. To date, several types of catalysts have been employed for CO_2_ conversion, including metal oxides, nitrides, sulfides, selenides, chalcogenides, and perovskite materials [12,13,14,15,16,17,18,19,20,21,22,23]. These materials have made significant progress, but many of them have several drawbacks, such as high-cost synthetic approaches, lengthy/complicated synthesis process, long-term instability, and less catalytic activity. While recently, perovskites have been significantly attracted as a better replacement for traditional semiconductors for the photocatalytic CO_2_ reduction process due to their extraordinary optoelectronic properties and cost-effectiveness [24,25].

Typically, perovskite materials are indicated with the chemical formula ABX_3_ [26,27]; here, A site occupies large size cation (e.g., Cs^+^, Rb^+^, methylammonium), and B sites are occupied by small size cation (e.g., Pb^2+^, Sn^2+^). At the same time, X (e.g., O^2−^, Br^−^, Cl^−^, I^−^) holds an anion that bonds to both A and B. According to the crystallographic perspective, the ideal perovskite structure is cubic and unbending; however, most perovskites are generally distorted. Based on the perovskite elements, the properties such as chemical stability, bandgap energies, optical stability, and crystal structure of the catalyst can be tunable [28]. In 1978, Hemminger et al. first-time observed photosynthetic reaction using SrTiO_2_ perovskite materials for the conversion of CO_2_ into CH_4_ in the presence of gaseous water and CO_2_ [29]. The SrTiO_2_ (111) crystalline phase with Pt foil was active for the CO_2_ conversion. The Pt foil was responsible for the adsorption of the CO_2_ on the metal faces, but the limitation was metal poisoning. In the case of SrTiO_3_, the regeneration of the Ti^3+^ after the oxidation reaction in water, surface properties, and crystallite size play a key role. Afterward, SrTiO_3_, Sr_2_TiO_4_, NaNbO_3_, and H_2_SrTa_2_O_7_ have been documented for the CO_2_ reduction process [30,31,32,33]. Luo et al. observed the relationship between the surface structure of SrTiO_3_ and photocatalytic activity for CO_2_ photoreduction [30]. The SrTiO_3_ was treated with the etching process and functionalized with the OH group. The SrO-terminated surface exhibits nucleophilicity, which allows CO_2_ adsorption, and TiO_2_-terminated is electrophilic. The effect of the electronic properties also differs from these two surfaces; the Sr 4d orbital conduction band level is more negative than the Ti 3d orbital. Though in the case of SrO-terminated or Sr(OH)_2_-decorated allowed the highest CO_2_ fixation than the Ti-rich surface, but the photoreduction activity was low. Because of the produced surface, molecules were attached to the weakly active Sr ions and exhibited lower reactivity. The Ti-rich catalyst showed the highest activity because the active Ti-edges shifted light absorption in the visible region. Therefore, these studies showed that the properties of perovskites, especially structural flexibility, will be of great interest to study as efficient materials for large-scale photocatalytic applications.

Among the various perovskite materials, halide perovskites have been successfully emerging as an efficient catalyst due to their extraordinary properties like cost-effectiveness, easy synthesis process, visible light absorption, high CO_2_ adsorption surface area, surface disorders for charge trapping, and tunable structure [34,35,36,37,38,39,40,41,42]. There are two classes of materials in the halide perovskites: (1) Lead-based halide perovskites and (2) lead-free halide perovskites. Most of the lead halide perovskites offer a narrow bandgap as compared to traditional semiconductors. The hybrid composites of lead halide perovskites with other semiconductors/materials can efficiently enhance the yield and stability of the CO_2_ conversion. Later, however, few more studies have been reported on lead-free halide perovskites to avoid toxicity. Therefore, lead and lead-free halide perovskites’ recent development towards reducing CO_2_ in different materials has fascinated us. This review would offer a thorough look into the recent success of optimal halide perovskites for the CO_2_ photoreduction application.

## 2. Fundamentals of Photocatalytic CO_2_ Reduction

The process of thermal catalysis is the easiest option for CO_2_ reduction. Still, it is not environmentally appropriate due to an endothermic reaction so that the input energy must be high [43]. As we already knew, nature is doing the same process that converts CO_2_ to hydrocarbons by the photosynthesis process. So why not artificial photosynthesis can help to solve the problem of CO_2_ reduction? The process of CO_2_ reduction begins after the chemisorption of the CO_2_ molecules on the catalytic surface. Therefore, the CO_2_ reduction process’s high efficiency can be achieved by employing high surface area catalysts to maximize CO_2_ molecules’ adsorption on the catalyst’s surface. As we know, CO_2_ is a highly stable molecule, therefore to break the C-O bonding, it requires 750 kJ mol^−1^ of the bond dissociation energy, which is approximately 54% higher than C-H bond dissociation energy (411 kJ mol^−1^). To reduce CO_2_ molecules, the conduction and valence band potentials of particular catalysts should be above and below the standard redox potentials of the products, respectively. Thus, to convert the CO_2_ into any product, it is necessary to cross the high energy of the activation barrier for forwarding reaction [44]. In the conversion of CO_2_, a single-electron transfer is practically inconceivable to produce CO_2_^●−^ due to the necessity of high redox potential of −1.90 V vs. NHE (see Table 1). Therefore, as a single electron process is practically challenging, the proton assisted CO_2_ reduction process is thermodynamically feasible to form various products. Depending upon the concentration of available electrons and protons, the CO_2_ reduction proceeds into the formation of various products; for instance, CO evolution can be achieved by contributing 2e^−^ and 2H^+^ at the redox potential −0.53 V (pH = 7). Consequently, an increase in the reduction potential concerning the number of electrons, converting CO_2_ to ethane, desires the 14e^−^ and 14H^+^. In recent days, there have been several excellent reviews based on the fundamentals of photo-reduction of CO_2_ [45,46,47,48,49,50]; thus, readers may turn to these reviews for more details. Our review article focuses on the fabrication of lead and lead-free halide perovskites’ and their composites, the effect of solvents, and other parameters to improve the catalytic performance towards the CO_2_ conversion process.

## 3. Lead Halide Perovskites for Photocatalytic CO_2_ Reduction

So far, organic–inorganic lead halide perovskites (LHPs) have been well established for photovoltaic applications with high power conversion proficiency [25]. Later, they have been successfully used for various applications, including photodetector, laser, LED, thermoelectric, and piezoelectric [51,52]. Recently, Zhu et al. reported LHPs for organic synthesis that have fundamental significance in drug production [53]. The high efficiency of LHPs was mainly attributed to the properties that are very suitable for photocatalytic applications, such as high absorption coefficient, greater defect tolerance, superior photogenerated charge-carrier lifetime, and high carrier mobility [54]. In recent years, LHPs have also been used as photocatalytic materials for various applications like hydrogen evolution reaction [55,56], organic pollutant degradation [57,58], and alkylation of aldehyde [41,51]. In addition, they have been proved the best materials for oxygen evolution reactions; thus, water can be used as an electron source for the photoreduction of CO_2_ by preventing the use of sacrificial agents [59]. Thus, the introduction of halide perovskites for CO_2_ photoreduction has made substantial progress in the field of catalysis. These materials possess poor water stability due to the ionic nature of the LHPs. Numerous attempts have been made to boost the catalytic activity and stability of LHPs towards photocatalytic CO_2_ reduction. In this contribution, Wu et al. encapsulated methylammonium lead iodide quantum dots (QDs) in iron-based metal–organic frameworks (MOFs) and successfully utilized them for CO_2_ photoreduction [60]. They have used Fe-porphyrin-based MOFs to increase the water stability of perovskite. Fe act as an active catalytic site for CO_2_ photoreduction, which also suppresses the charge recombination and effectively enhances the charge transportation. The optimized samples of MAPbI_3_ with Fe-porphyrin MOF PCN-221(Fe_x_) exhibited a total hydrocarbon yield of 1559 µmol g^−1^ combined for CO and CH_4_ production. As the concentration of Fe in PCN-221 (Fe_x_) increases, the improvement in CO and CH_4_ formation was observed. The catalyst showed excellent stability over 80 h, which was much higher than pristine halide perovskite QDs. Remarkably, water was used as a sacrificial agent with ethyl acetate, which acts as an electron source for the CO_2_ reduction reaction. The light-harvesting efficiency of MAPbI_3_ and fast electron transfer from MAPbI_3_ to Fe were attributed to high catalytic activity.

**Figure 1:**
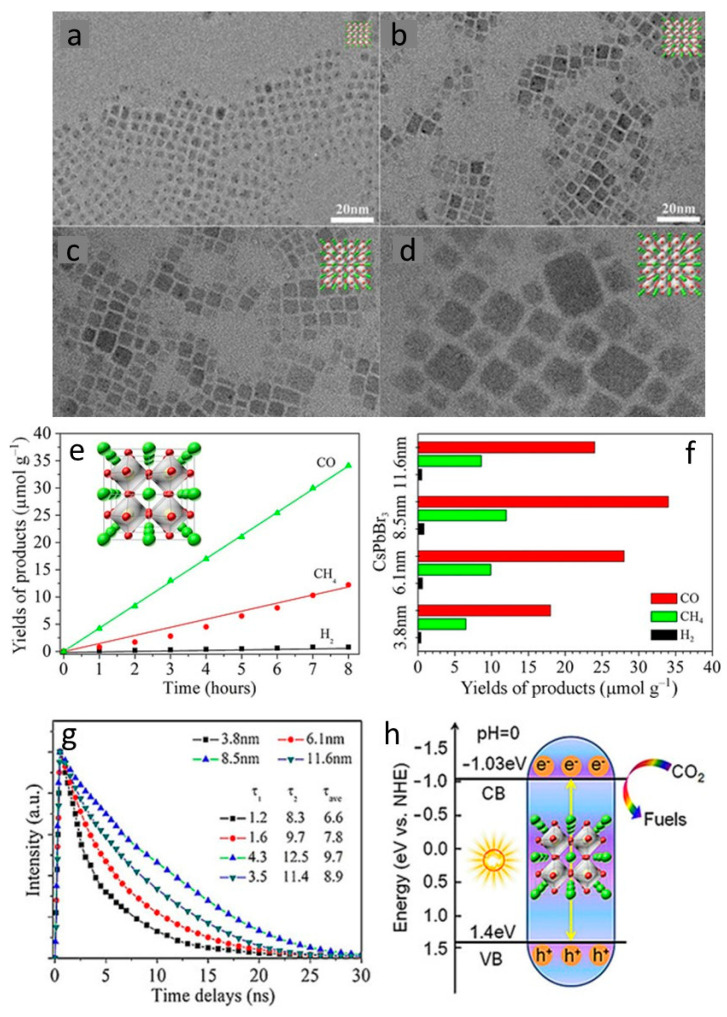
(**a–d**) CsPbBr_3_ quantum dots (QDs) TEM images showing the difference in particle size, (**e**) photocatalytic CO_2_ reduction using an optimized sample of CsPbBr_3_, (**f**) particle size effect on the CO_2_ reduction activity, (**g**) Time-resolved photoluminescence (TRPL) decay of different samples, and (**h**) band diagram showing mechanism of CO_2_ reduction to chemical fuels. Reproduced from [61], with permission from John Wiley and Sons, 2017.

Apart from this exceptional research, most methylammonium halides have been documented for hydrogen production. Previous studies have shown that MAPbI_3_ is unstable in humid conditions; thus, other halide perovskites have been intended for CO_2_ photoreduction [61]. Subsequently, major advances have been made in the field of research with cesium lead halide perovskites as a novel and most common catalyst for CO_2_ reduction in recent years. Most of the inorganic Cs-based perovskites possess suitable valence and conduction band potentials for the CO_2_ reduction reaction. To this end, Hou et al. suggested a controlled synthesis of colloidal QDs of CsPbBr_3_ and investigated the size-dependent CO_2_ photoreduction into CH_4_, CO, and H_2_ [61]. Using less costly inorganic precursors and oleylamine/oleic acid as a surface ligand, the QDs were synthesized using a simple solution process. These colloidal QDs display the tunable particle size due to the quantum confinement effect at different temperatures, as shown in Figure 1a–d. The optical properties of CsPbBr_3_ showed a significant impact on photocatalytic efficiency (Figure 1e,f). During the photocatalytic reaction, the average electron yield for CO_2_ reduction was 20.9 μmol g^−1^. A Time-resolved PL study signifies that an optimized catalyst has the most extended lifetime responsible for enhanced catalytic activity (Figure 1g). The band alignment of the valence and conduction band were well matched for CO_2_ reduction and water oxidation leading to the formation of CO, CH_4_, and H_2_ (Figure 1h). Therefore, such QDs can be used as inexpensive catalysts for catalytic applications.

Mixed halide perovskites, i.e., a mixture of the cation (MA^+^, Cs^+^) and anion (Cl^−^, I^−^, Br^−^), can further increase the efficiency of catalytic reaction towards CO_2_ reduction. Tuning the halide ratio can change the structural combination of perovskites that significantly impact catalytic behavior. Thus, Su and co-workers reported a low-cost, cubic phase CsPb(Br_0.5_/Cl_0.5_)_3_ perovskite with varied ratios of Br and Cl and studied for photocatalytic CO_2_ reduction [62]. The controlled synthesis of mixed halide perovskite was carried out by the hot injection method. Mixed halide perovskite, i.e., CsPb(Br_x_/Cl_1−x_)_3,_ offers excellent absorption in the visible region ranging from 400–700 nm, relatively broader than distinctive semiconductors like TiO_2_ and ZnO. The calculated band edges were quite suitable for CO_2_ reduction potentials of CO and CH_4_, with the bandgap ranging from 2.33 to 2.98 eV for CsPb(Br_x_/Cl_1−x_)_3_ samples. While in the emission spectra, the gradual shifting and quenching of pristine CsPbBr_3_ from 517 to 413 nm was observed as the amount of Cl increases, which is attributed to the improved electron separation. The authors performed a CO_2_ reduction test in ethyl acetate. The increased CO and CH_4_ formation was found with an increase in the Cl concentration, and the optimized sample achieved much higher catalytic activity than the pristine CsPbBr_3_ and CsPbCl_3_ perovskites. The time-dependent CO_2_ reduction rate for the optimized catalyst demonstrated strong stability over the 9 h for CH_4_ and CO evolution. The increased activity and stability of mixed halide perovskite were attributed to the controlled ratios of Br and Cl. 

**Figure 2:**
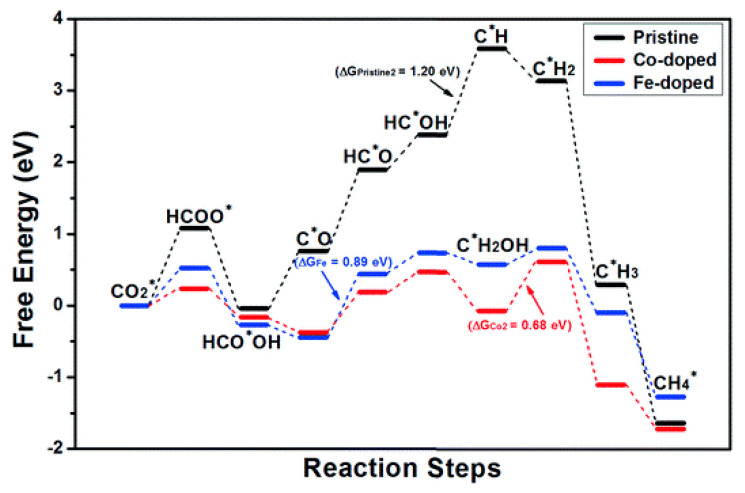
The free energy diagram showing the pathway of CO_2_ reduction on pristine and Co/Fe doped CsPbBr_3_. Reproduced from [63], Royal Society of Chemistry, 2019.

It is well known that pristine semiconductor/perovskites suffer from poor catalytic activity and stability due to the rapid recombination of photogenerated charges or lack of suitable optical absorption, or ineffective CO_2_ adsorption. Therefore, several studies have been documented with metal doping for cesium halide perovskites to boost catalytic activity, stability, and selectivity. In order to attain these distinct characteristics, it is crucial to consider the mechanism of product formation and the pathway of CO_2_ reduction over the catalyst. Tang et al. theoretically studied the effect of metal doping in CsPbBr_3_ on product selectivity by employing DFT calculations (Figure 2) [63]. The model used for this analysis has considered the chemical potential of an electron and proton is equal to half of the hydrogen gas phase at standard pressure. The first step of CO_2_ reduction formed the HCOO*, and further adsorption of the excessive proton on the oxygen atom leads to HCO*OH formation. The HCO*OH is a spontaneous reaction; however, dissociation of HCO*OH to CO and H_2_O is the nonspontaneous reaction in the case of pristine-CsPbBr_3_. After Co and Fe doping, the catalytic activity was improved and showed a downhill reaction. The numerical values of Gibbs free energy indicated that Co and Fe doping increases the rate of chemical reaction approximately two times for catalytic activity than pristine-CsPbBr_3_ material. Pristine CsPbBr_3_ was initially examined to reduce CO_2_, and the findings of the free barrier energy showed that CsPbBr_3_ is inactive for the evolution of any hydrocarbons. While Fe and Co-doped CsPbBr_3_ were investigated for selective formation of CH_4_, these metals have great potential to break the O-C-O bond. The resultant activity and selectivity towards CH_4_ formation were ascribed to the successful adsorption and activation of CO_2_**^●^** on the doped CsPbBr_3_. After this, Pradhan and co-workers practically explored Fe (II)-CsPbBr_3_ for selective CH_4_ evolution via CO_2_ photoreduction [64]. Doping of Fe (II) replaces Pb (II) in the CsPbBr_3_ lattice, and an increased CH_4_ selectivity was observed over the increment of Fe (II) concentration; however, pristine CsPbBr_3_ forms CO. Later, another study by Su et al. showed that Mn^2+^ substitution to perovskite could significantly improve the optical and thermal properties of halide perovskites [65]. They observed that Mn-doped cesium lead halide (Br/Cl) perovskite showed more than 14 times improvement in catalytic performance than pristine catalysts. The Mn-CsPb(Br/Cl) exhibited the catalytic towards CO and CH_4_ formation with a yield of 1917 µmol g^−1^ and 82 µmol g^−1^, respectively. Other metals such as Fe and Co have been reported as ideal dopants with cesium perovskite, and improved water sustainability was reported [63]. For instance, Fe (II) doped CsPbBr_3_ and Co-doped CsPbBr_3_ have been reported for the evolution of CO and CH_4_ by Prahan et al. and Lu et al., respectively. Particularly, Fe doped CsPbBr_3_ predominantly forms CH_4_ while pristine nanocrystals (NCs) are selective towards CO formation [64].

Afterward, more outcomes of metal confined halide perovskites are reported. Lu and co-workers’ study showed that Co-doped CsPbBr_3_/Cs_4_PbBr_6_ combination improved catalytic performance for CH_4_ and CO evolution [59]. Co doping to perovskite has two benefits; first, it produces surface trap states due to the presence of Co^2+^ and extends the lifetime of photogenerated charges. Second, it broadens the adsorption of CO_2_* intermediate to the catalytic surface. Remarkably, they have employed hexafluorobutyl methacrylate to improve NC’s stability and dispersity in an aqueous medium. Then, in this class of materials, Zhang and coworkers reported Co-doped CsPbBr_3_ embedded in the matrix of Cs_4_PbBr_6_ (a surface protector) [66]. The reason behind such protection of Cs_4_PbBr_6_ was to improve the stability of ligand-free CsPbBr_3_ perovskite. As a result, the optimized Co-doped perovskite composite exhibited excellent CO_2_ reduction towards CO formation with 1835 µmol g^−1^ in 15 h. In combination with the acetonitrile/water solution, the methanol was used as a hole scavenger to improve the catalytic activity towards CO_2_ reduction. Interestingly, Cs_4_PbBr_6_ does not take part in the redox reaction due to its unsuitable band potentials/alignment; however, CsPbBr_3_ takes part in the CO_2_ reduction process and triggers the catalytic activity towards CO formation. After these reports, Pt has also been successfully used as a co-catalyst for CsPbBr_3_ [67]. This study stated the solvent effect, where it was observed that acetate is the most potent solvent, which provides a stable atmosphere for perovskite to conduct the CO_2_ reduction process. The optimized Pt loaded catalyst demonstrated an electron consumption rate of 5.6 µmol g^−1^ h^−1^ in ethyl acetate. In another study of Zhu and co-workers, the water-stable CsPbCl_3_ was documented with Mn and Ni doping. They illustrated the significance of the Pb-rich surface, which extends the lifetime of PL to increase the catalytic activity [68]. By interpenetrating solid–liquid, the synthesis of surface Pb enriched CsPbCl_3_ was accomplished by allowing the water to drain through the CsPbCl_3_ layer originating with Cs^+^ and Cl^−^. These Cs^+^ and Cl^−^ inhibit the decomposition of CsPbCl_3_ and increase the PL lifetime. Therefore, Ni-doped Pb-rich CsPbCl_3_ QDs displayed superior CO_2_ reduction behavior against CO evolution with a rate of 169.37 μmol g^−1^ h^−1^.

In addition to metal doping/deposition, perovskites are combined with supporting material to increase light absorption and charge separation. Among various materials, graphene can be a good choice as a support material for perovskites due to its well-known surface, optoelectronic, and physicochemical properties [69,70], which ultimately prolongs the electron/hole pair’s lifetime and makes perovskite ideal for photocatalytic CO_2_ conversion. Hence, Xu et al. fabricated CsPbBr_3_ QDs/graphene oxide (GO) composite by a simple precipitation process for photoconversion of CO_2_ in a nonaqueous medium [71]. The CO_2_ reduction reaction was conducted in a Pyrex bottle using ethyl acetate as a solvent. The use of ethyl acetate has two benefits; (i) it stabilizes CsPbBr_3_ due to its moderate polarity; and (ii) it increases the solubility of CO_2_ even more than in water. The presence of GO provides additional electron transfer from CsPbBr_3_ to GO; therefore, it was found that the CsPbBr_3_/GO composite exhibited improved catalytic activity compared to pristine CsPbBr_3_ QDs. The catalyst displayed stability over 12 h, and no phase change of CsPbBr_3_ was observed during the catalytic reaction. Excited electrons from CsPbBr_3_ quickly transfer to the GO sheet, suppress the electron-hole pair’s recombination, and increase catalytic activity.

Shortly after, few more studies have been reported in combination with graphene. For instance, Eslava and the group reported a surfactant-free synthesis of CsPbBr_3_ NCs [72]. They have synthesized CsPbBr_3_ on a gram scale by employing the simple mechanochemical process to get the different morphology of NCs, including nanorods, nanosheets, and nanospheres. To improve the catalytic efficiency, CsPbBr_3_ was further combined with Cu-RGO by the mechanochemical process. The CsPbBr_3_ nanosheets with Cu-RGO achieved 12.7, 0.46, and 0.27 μmol g^−1^ h^−1^ of CH_4_, CO, and H_2_ evolution rates after CO_2_ reduction. The catalyst composed of CsPbBr_3_-Cu-RGO achieved 1.10% apparent quantum efficiency and showed excellent stability over three consecutive runs. Such an alternative method of large-scale synthesis is notable and essential for advancing the photocatalytic technology practically. Later, Wang and co-workers demonstrated the effectiveness of RGO sheets combined with Cs_4_PbBr_6_ for CO_2_ reduction [73]. They revealed that the dual nature of the RGO is responsible for increased catalytic activity and stability, (i) the defect-induced RGO efficiently traps the electron excited by Cs_4_PbBr_6_, and (ii) oxygen-deficient RGO adsorbs and stimulates CO_2_ molecule. In addition to these studies, Mu and co-workers described the ultrathin small-sized graphene oxide (USGO) as an electron mediator between CsPbBr_3_/α-Fe_2_O_3_ Z-scheme photocatalyst [74]. Intense contact between CaPbBr_3_/USGO through the Br-O-C bond; and USGO/α-Fe_2_O_3_ via the C-O-Fe bond accelerates the transfer of electrons through the Z-scheme. As a result, such a Z-scheme combination achieved 9 times greater catalytic efficiency compared to CsPbBr_3_ NCs. Later on, such a Z-scheme heterojunction of CsPbBr_3_ QDs was also reported with Bi_2_WO_6_ nanosheet [75]. Therefore, the Z-scheme combination then performs efficient charge separation across the closely connected interface and enhances the catalytic activity. 

**Figure 3:**
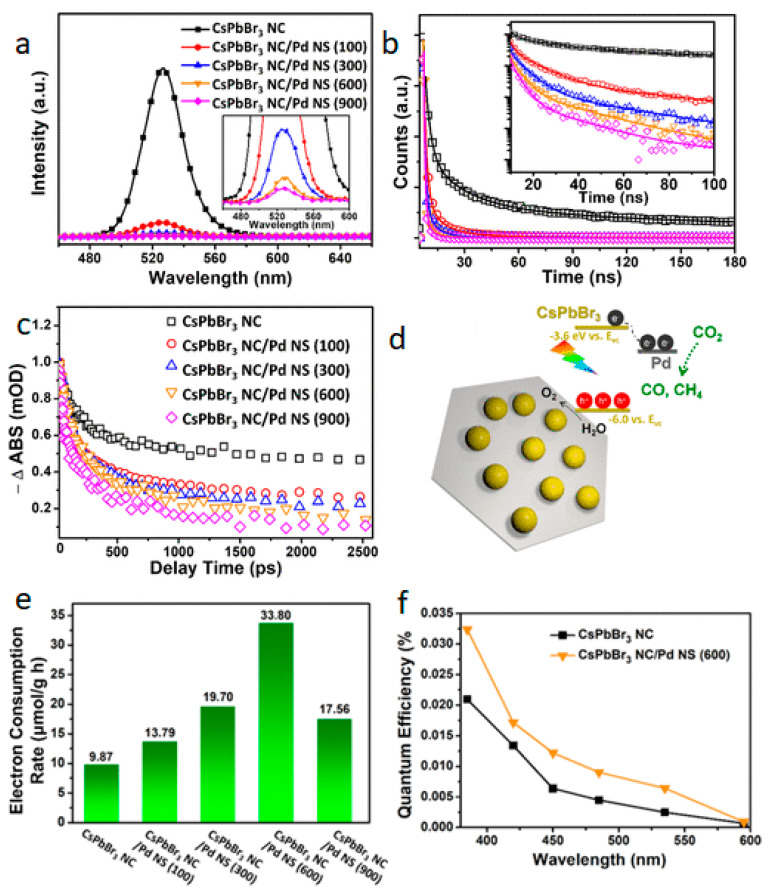
The comparative (**a**) PL spectra, (**b**) PL decay, (**c**) transient absorption kinetic plots (at an excitation wavelength of 400 nm) among CsPbBr_3_ NC and composite samples, (**d**) schematic illustration and band alignment of CsPbBr_3_/Pd composite for CO_2_ reduction, (**e**) photocatalytic CO_2_ reduction performance, and (**f**) quantum efficiency of different samples. Reproduced from [76], with permission from Royal Society of Chemistry, 2019.

Recently, 2D materials have grabbed huge attention as the best supporting materials in many applications. A halide perovskites/2D composite materials strategy can achieve an efficient charge transfer and reduced electron-hole recombination among the perovskite-based photocatalysts. Xu et al. produced the CsPbBr_3_/Pd Schottky junction in 2018 and studied the enhanced consumption rate of electrons for photoreduction of CO_2_ [76]. For this analysis, CsPbBr_3_ NCs were deposited on Pd nanosheets under atmospheric conditions using a simple technique on a glass substrate, and the photocatalytic reduction of CO_2_ to CO/CH_4_ was studied. The charge transport and charge carrier dynamics among the composite were studied by PL and fs-TAS (femtosecond transient absorption spectroscopy). The PL quenching of 0.5–8.6% in the CsPbBr_3_/Pd composite was observed compared to the pristine sample (Figure 3a). Likewise, the decreased PL decay (Figure 3b) of the composite was observed in TRPL analysis with an average lifetime of 2.71–13.38 ns; however, such a decay lifetime for CsPbBr_3_ was measured to be 52.03 ns. Furthermore, a similar trend was observed in the fs-TAS analysis (Figure 3c), where a large decrease in peak intensity of composite than the pristine sample was observed. These findings indicate that the recombination rate of the electron-hole in CsPbBr_3_ was reduced by integrating it over Pd nanosheets, which consciously promotes the process of CO_2_ reduction to form CH_4_ and CO (Figure 3d). Remarkably, the Schottky contact between CsPbBr_3_/Pd composite increases the electron consumption rate up to 33.80 µmol g^−1^ (for CH_4_ and CO evolution), 2.43 times higher than the pristine CsPbBr_3_ with the improved quantum efficiency (Figure 3e,f). Therefore, a combination of halide perovskite with 2D materials proved to be a good strategy for the photocatalytic CO_2_ reduction reaction.

**Figure 4:**
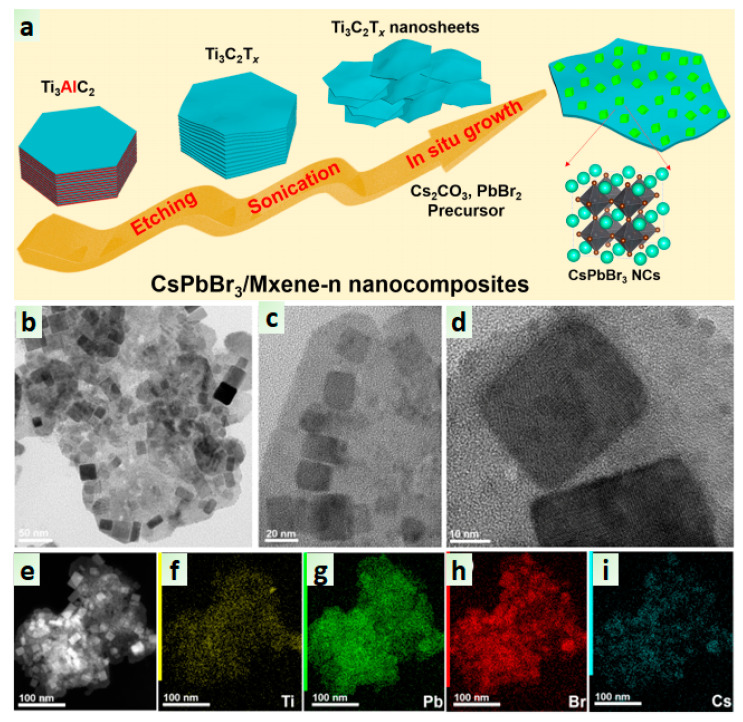
(**a**) An in−situ synthetic procedure for CsPbBr_3_/MXene, (**b**−**d**) TEM and HR-TEM images of CsPbBr_3_/MXene-20 composite and (**e**−**i**) EDX elemental mapping for the respective elements. Reproduced from [77], with permission from American Chemical Society, 2019.

Later on, Liu et al. introduced a functional CsPbBr_3_/MXene nanocomposite for CO_2_ reduction to CO and CH_4_ under visible light [77]. The consistent growth of CsPbBr_3_ on exfoliated MXene_−n_ (n = 10, 20, 30, 40, and 50, a different amount of MXene) nanosheet was achieved by an in-situ method, as shown in Figure 4a. The etching of Ti_3_AlC_2_ was carried out by using the HCl-HF solution to form a Ti_3_C_2_T_x_ nanosheet. Then, the final nanocomposite was obtained by the exfoliation of multilayered Ti_3_C_2_T_x_ and in-situ growth of CsPbBr_3_ on Ti_3_C_2_T_x_ nanosheets. The dispersion of cubic CsPbBr_3_ with an average size of 25 nm on MXene nanosheets was observed in the TEM images (Figure 4b–d), and the presence of Ti, Pb, Br, and Cs confirms the constant growth of perovskite NCs on MXene nanosheet (Figure 4e–i). As expected, the PL and TRPL quenching in composite compared to pristine perovskite confirm the efficient charge transfer among the interface of CsPbBr_3_/MXene. The photocatalytic CO_2_ reduction test was carried out under light irradiation using ethyl acetate solvent towards CO and CH_4_ formation with a rate of 26.32 and 7.25 μmol g^−1^ h^−1^, respectively. Therefore, such perovskite/2D composites can be used as an efficient catalyst for photocatalytic applications.

Construction of perovskites/semiconductor heterojunction is advantageous to improve the optoelectronic or photochemical properties of catalytic systems. As well known, when two semiconductors with different band potentials combine, heterojunction forms at the interface of particular semiconductors and facilitate the charge separation process. Among various candidates, TiO_2_ was proven to be an excellent semiconducting material all over the years for photocatalytic applications. In this aspect, Xu et al. in 2018 reported an amorphous TiO_2_ encapsulated CsPbBr_3_ composite with enhanced catalytic efficiency [78]. The improved separation of the charge between CsPbBr_3_ and TiO_2_ was shown by the decay of the PL and TRPL, where dramatic quenching of radiative recombination was observed. Moreover, the fs-TAS analysis revealed that the decreased electron-hole recombination improves the charge separation efficiently among the composite. As a result, the CsPbBr_3_/amorphous TiO_2_ composite demonstrated 6.5 times better photoelectron intake during the CO_2_ photoreduction and stability over 30 h. The high selectivity towards CH_4_ formation was attributed to the perfect combination of CsPbBr_3_ and amorphous TiO_2_. The electron generated by CsPbBr_3_ accumulates on TiO_2_ to break the dynamic barrier and therefore fast-track the rate of CH_4_ formation. Thus, such a study helps to understand the surface modification of halide perovskites for various applications. 

**Figure 5:**
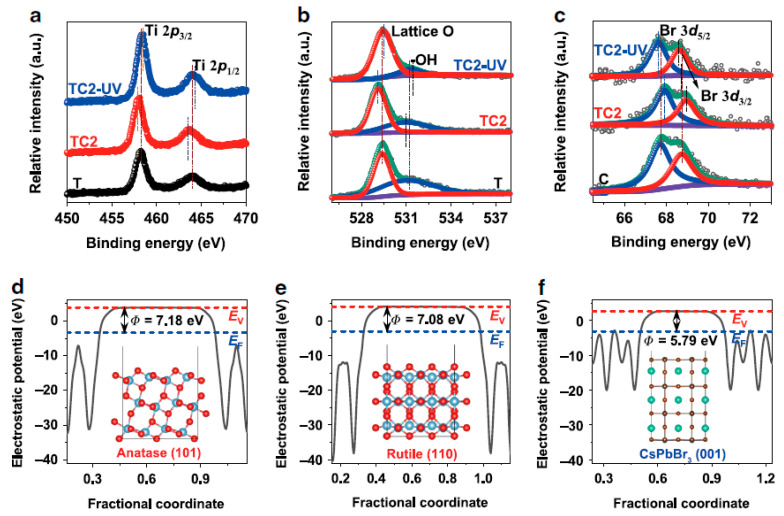
(**a**−**c**) Ex-situ and in-situ XPS of TiO_2_ (T) and TiO_2_/CsPbBr_3_ (TC2) samples (TC2-UV is in-situ XPS under UV light irradiation), and electrostatic potentials for (**d**) anatase TiO_2_ (101), (**e**) rutile TiO_2_ (110) and (**f**) CsPbBr_3_ (001) facets. Reproduced from [79], with permission from Springer Nature, 2020.

Afterward, in 2020 Yu and co-workers developed a self-assembled CsPbBr_3_ QDs/TiO_2_ nanofibers, an S-scheme heterojunction hybrid for CO_2_ reduction under the irradiation of UV-Vis light [79]. The electron microscopy results revealed that CsPbBr_3_ QDs were uniformly distributed on TiO_2_ nanofibers. Density functional theory (DFT) and experimental studies were combined to comprehend the interfacial charge transfer among the composite. The chemical states of pristine and composite samples were explored by in-situ and ex-situ XPS analysis. As shown in Figure 5a,b, the Ti 2p peak of Ti^4+^ ions, O 1s of lattice oxygen, and surface -OH group are present in all the samples. However, in the in-situ measurement, the binding energies (BE) of Ti 2p and O 1s peaks shifted towards higher BE than ex-situ spectra. A similar observation but opposite peak shift was observed in the Br 2d peak, mainly attributed to an electron transfer from CsPbBr_3_ to TiO_2_ (Figure 5c). Such electron transfer is responsible for constructing the S-scheme heterojunction among TiO_2_/CsPbBr_3_, which efficiently separates the photogenerated charges to promote CO_2_ reduction. The electron transfer from CsPbBr_3_ to TiO_2_ was further validated by work function values calculated from the energy difference of vacuum and fermi levels, as shown in Figure 5d–f. Due to the lower Fermi level of TiO_2_ than CsPbBr_3_ QDs (work function (ϕ) of CsPbBr_3_, 5.79 eV and TiO_2_, 7.18 or 7.08 eV), the electron flow would be favorable from CsPbBr_3_ to TiO_2_ for enabling the phases at the similar Fermi level and created an internal electric field at the interface of TiO_2_/CsPbBr_3_. A similar observation was observed in DFT calculations. Therefore, such an improved electron transfer is responsible for the enhanced catalytic performance of hybrid (9.02 µmol g^−1^ h^−1^) than the pristine CsPbBr_3_ and TiO_2_ (4.94 and 4.68 µmol g^−1^ h^−1^), respectively, towards CO formation. The improved CO_2_ adsorption on CsPbBr_3_ QDs and S-scheme heterojunction formation was ascribed to superior photocatalytic activity.

Next, 3D structures, e.g., 3D microporous graphene, exhibited possible support material for catalytic reactions [80,81]. Such a 3D network structure can provide more CO_2_ reactive sites and provide fast charge transport across multidimensional networks. Kaung and co-workers fabricated the hierarchical ternary nanocomposite of CsPbBr_3_ with ZnO nanowire/3D graphene through a multi-step process for photocatalytic CO_2_ reduction [81]. First, in-situ 1D ZnO/2D RGO macropores with a high specific surface area were fabricated on a film. Then, as-prepared CsPbBr_3_ was used for the synthesis of ternary composite through the centrifugation cast method. SEM and TEM images revealed that the CsPbBr_3_ NCs are well decorated in the ZnO nanowires over RGO. Similarly, optoelectronic and surface properties showed improved light harvesting in the visible region and improved CO_2_ adsorption on the catalyst’s surface, respectively. As a result, the ternary composite exhibited 52.02 µmol g^−1^ h^−1^ of CH_4_ evolution (96.7% selectivity) along with CO formation. The electron pathway for CO_2_ reduction was achieved via CsPbBr_3_ to 1D ZnO to 3D RGO.

Apart from metal oxides, CsPbBr_3_ can also be anchored with g-C_3_N_4_ due to its superior properties, such as visible-light active, tunable band potentials, and rich active surface area [82,83]. The combination of CsPbBr_3_ and g-C_3_N_4_ facilitates efficient charge transport through their closely connected interface. In this regard, Xu et al. attached CsPbBr_3_ QDs to amino-functionalized g-C_3_N_4_ nanosheet through N-Br bonding [83]. The 20 wt.% contained QDs anchored on g-C_3_N_4_ achieved a superior photocatalytic CO_2_ reduction towards CO formation with a rate of 149 μmol h^−1^ g^−1^ in acetonitrile/water solvent. The surface functionalization of g-C_3_N_4_ with abundant NH_x_ was shown to help build a bridge between g-C_3_N_4_ and CsPbBr_3_. Such N-Br bonding was confirmed by XPS analysis, which is responsible for the enhanced charge separation and decreased electron-hole recombination rate. Resulting, the composite showed 15- and 3-fold improved catalytic activity than pristine CsPbBr_3_ QDs and g-C_3_N_4_. Later, in 2019 a similar study was reported by Zhang and co-workers for CsPbBr_3_/g-C_3_N_4_ containing TiO species (TiO-CN) [84]. The well-defined composite of CsPbBr_3_@TiO-CN was able to undergo CO_2_ reduction to produce a 129 μmol g^−1^ of CO under 10 h visible light irradiation. The interaction among CsPbBr_3_ and g-C_3_N_4_ was established via N-Br and O-Br, reducing the recombination of the electron/hole pair. The electrons generated in the conduction band of CsPbBr_3_ transfer to TiO-CN nanosheet and react with adsorbed CO_2_ molecules. At the same time, water oxidation was carried out by holes accumulated at the valence band of CsPbBr_3_.

**Figure 6:**
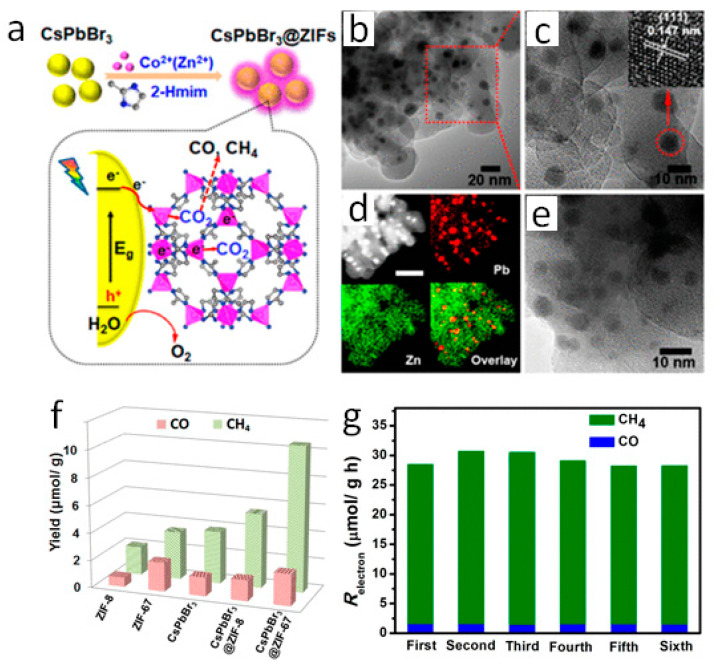
(**a**) Schematic illustration of synthesis and utilization of CsPbBr_3_/ZIFs for CO_2_ reduction, (**b**,**c**) TEM of CsPbBr_3_@ZIF-8, (**d**) HAADF-STEM of CsPbBr_3_@ZIF-27, (**e**) TEM of CsPbBr_3_@ZIF-27, (**f**) CO_2_ reduction results for pristine and composite, and (**g**) Stability test for CsPbBr_3_@ZIF-67. Reproduced from [85], with permission from American chemical society, 2018.

The core-shell combinations have been reported as an alternative option to improve halide perovskite’s stability and activity towards CO_2_ reduction, where coating the surface of perovskite may also upsurge water stability. Hence, various materials, including metal oxides, polymers, silica, zeolites, and metal–organic framework (MOF), have been successfully utilized. These days, MOFs are widely employed for photocatalytic applications due to their unique properties such as high specific surface area, more catalytic active sites, and tunable structural flexibility. In this way, CsPbBr_3_@ZIF (zeolitic imidazolate framework) was reported for an efficient CO_2_ photoreduction [85]. In this study, CsPbBr_3_ was coated with the Zn-based metal-organic system ZIF-8 and the Co-based ZIF-67 by an in-situ approach that activates the CO_2_ molecule, as shown in Figure 6a. The HAADF-STEM images and elemental mapping confirmed the formation of the CsPbBr_3_@ZIF core-shell structure, as shown in Figure 6b–e. It has been reported that ZIF coating increases the stability of CsPbBr_3_ due to its weak hydrophobic nature. The gas-phase photocatalytic CO_2_ reduction with water vapor showed CH_4_ and CO evolution; and, the CH_4_ formation was increased with the increase in irradiation time and achieved 100% selectivity. The increased catalytic activity was demonstrated by composite with ZIF-67 achieving 10.53 μmol g^–1^ of CH_4_ evolution (Figure 6f). The electron consumption rate for ZIF-8 and ZIF-67 composites was 15.498 and 29.630 μmol g^–1^ h^–1^. Moreover, the CsPbBr_3_@ZIF catalyst showed stability for six consecutive cycles, which proves its excellent proficiency (Figure 6g). Therefore, such studies could lead to fabricated highly stable hybrid composites of perovskite materials. Later on, few more studies have been reported on a similar class of hybrid perovskites. For instance, Wang and co-workers successfully developed CsPbBr_3_ QDs/UiO-66(NH_2_) nano junction and employed it for visible-light-active CO_2_ reduction [86]. TEM images confirmed the construction of a nano junction between CsPbBr_3_ and UiO-66(NH_2_). The optimized catalyst was able to produce 98.57 μmol g^−1^ of CO and 3.08 μmol g^−1^ of CH_4_. Interestingly, the specific surface of pristine UiO-66(NH_2_) was 709.02 m^2^ g^−1^, which was more than the nanocomposite (465.68 m^2^ g^−1^); the catalyst exhibited much more catalytic activity than the bare samples. The catalyst’s reusability was reported for three cycles, proving its high chemical stability and photo resistivity. The suitable VB and CB potentials of CsPbBr_3_ and HOMO-LUMO of UiO-66(NH_2_) were well matched for CO_2_ reduction and water oxidation to generate H^+^ and O_2_, resulting to form CO. The photocatalytic reactions were carried out in ethyl acetate/H_2_O combination; therefore, H_2_ evolution could be possible. However, the absence of any co-catalyst restricts the H_2_ evolution and selectively produces CO more efficiently along with CH_4_.

Metal complexes have long been recognized for CO_2_ photoreduction due to their exciting characteristics such as high selectivity and CO_2_ conversion activity, and structural flexibility [87,88,89,90]. The conjugated structures of metal complexes are commonly employed as a multi-electron transporter in the catalytic process. Moreover, the structural flexibility of these materials proved beneficial for tailoring catalytic activity and product selectivity. Previously, metal complexes were employed for visible-light-driven CO_2_ reduction combined with various organic photosensitizers [91]. In the study of Kaung and co-workers in 2020, similar class of material anchoring CsPbBr_3_ with Re(CO)_3_Br(dcbpy) (dcbpy^¼^4,4′- dicarboxy-2,2′-bipyridine) complex has been reported [92]. The interface between Re-complex and CsPbBr_3_ was established through the carboxyl group, which is responsible for the fast electron transfer to boost the catalytic activity towards CO_2_ reduction. Hence, the optimized catalyst showed 23 times higher electron consumption rate than CsPbBr_3_ towards CO evolution. However, arduous synthetic procedures of photosensitizer or the use of precious metals restrict them for large-scale applications. Later, the combination of CsPbBr_3_ perovskite NCs with (Ni(tpy)), a hybrid transition metal complex, was developed by Gaponik et al. and cast-off for the conversion of photocatalytic CO_2_ into CO/CH_4_ [93]. The synthesis of the hybrid composite includes multi-steps, synthesis of (i) organic ligands of CsPbBr_3_, (ii) ligand exchange, and (iii) assembly of CsPbBr_3_-Ni(tpy) by immobilization. The charge transfer between the composite was confirmed by TRPL decay and transient absorption spectroscopy, where the electron transfer from CsPbBr_3_ to Ni(tpy) was observed. Thus, under the light irradiation, the catalyst undergoes CO_2_ reduction and achieved 1724 μmol g^−1^ of CO/CH_4_ formation. The catalytic activity was shown to be 26 times higher than the pristine CsPbBr_3_ and AQE of 0.23% for CO and CH_4_ evolution under monochromatic light (450 nm). Also, the catalyst showed stability over 16 h, and post catalytic analysis confirmed its high stability. Therefore, Ni(tpy) offers more catalytic sites for the CO_2_ molecule and improves the catalytic performance.

**Figure 7:**
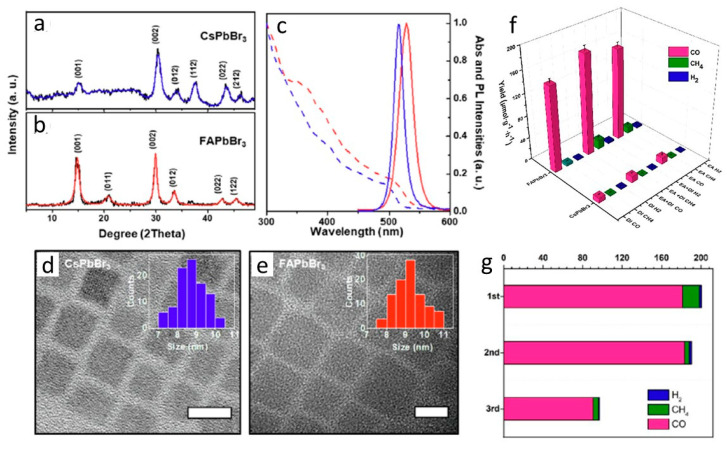
(**a,b**) The XRD patterns, (**c**) UV-Vis/PL spectra and (**d**,**e**) TEM images of FAPbBr_3_, and CsPbBr_3_, (**f**) Results of comparative photocatalytic CO_2_ reduction in FAPbBr_3_ and CsPbBr_3_, and (**g**) reusability test of FAPbBr_3_. Reproduced from [94], with permission from Elsevier, 2020.

After MAPbBr_x_ and CsPbBr_x_, Que and co-workers introduced a novel FAPbBr_3_ as an alternative option for traditional perovskites [94]. The synthesis of FAPbBr_3_ was carried out by a hot injection method, and the results were compared with CsPbBr_3_ synthesized similarly. As shown in Figure 7a–e, the XRD patterns and optical properties of as-prepared FAPbBr_3_ are almost identical to that of CsPbBr_3_, and they possess identical morphology with cubic shape. Despite, FAPbBr_3_ showed an enormous improvement in the CO evolution (main product) under the CO_2_ photoreduction compared to CsPbBr_3_, achieving 181.25 μmol g^−1^ h^−1^, which was ≈17 times greater than CsPbBr_3_ (Figure 7f). The significant cyclic stability was observed in FAPbBr_3_, preserving more than 165 μmol g^−1^ h^−1^ of CO evolution after three cycles (Figure 7g). Such high catalytic efficiency in FAPbBr_3_ was due to the improved lifetime of 7003 ps compared to CsPbBr_3_ with 956 ps.

## 4. Lead-Free Halide Perovskites

Throughout the years, Pb-based perovskites have been proved as the most efficient materials for photocatalytic CO_2_ reduction applications due to their excellent photophysical properties. Nevertheless, Pb perovskites’ high toxicity may restrict large-scale applications in the near future [95]. Li and colleagues’ recent research revealed that the biological impact of Pb-perovskite is unsafe, which shows that Pb could reach the human food chain by plants from perovskites leakage into the ground [96]. Lead exposure can cause a severe problem to human health, including nausea, clumsiness, muscle weakness, and clouded consciousness [97,98]. Therefore, eliminating Pb from perovskite structure should be the primary concern to use them for long-term applications [99,100]. To this end, numerous attempts are being made to replace Pb from halide perovskite structure with other potential candidates, including Sn, Sb, Bi, Cu, In, and Pd. In this contribution, Chu et al. published a review article on lead-free halide double perovskites for various applications covering photodetector, X-ray detector, LEDs, solar cells, and photocatalysis [98]. To date, a limited number of studies were carried out on Pb-free halide perovskites for photocatalytic CO_2_ reduction [101]. 

**Figure 8:**
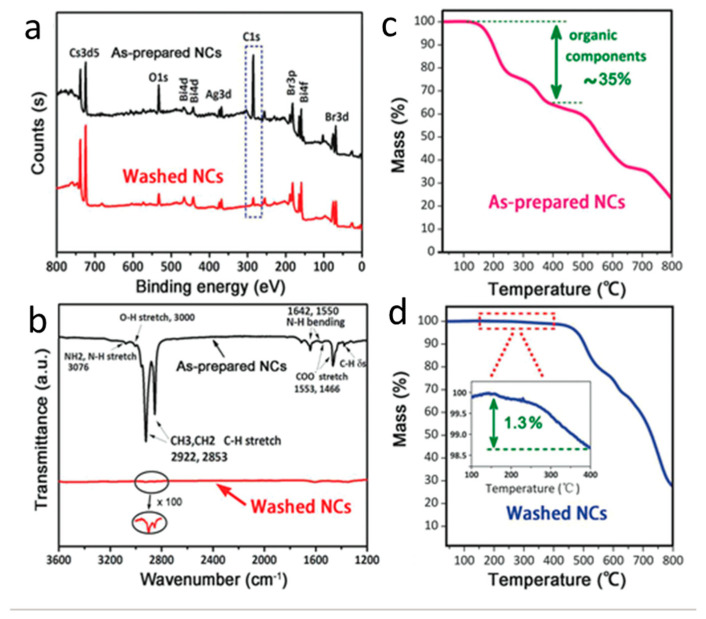
(**a**) XPS, (**b**) FTIR, and (**c**,**d**) TGA analysis of Cs_2_AgBiBr_6_ before and after the washing. Reproduced from [104], with permission from John Wiley and Sons, 2018.

**Figure 9:**
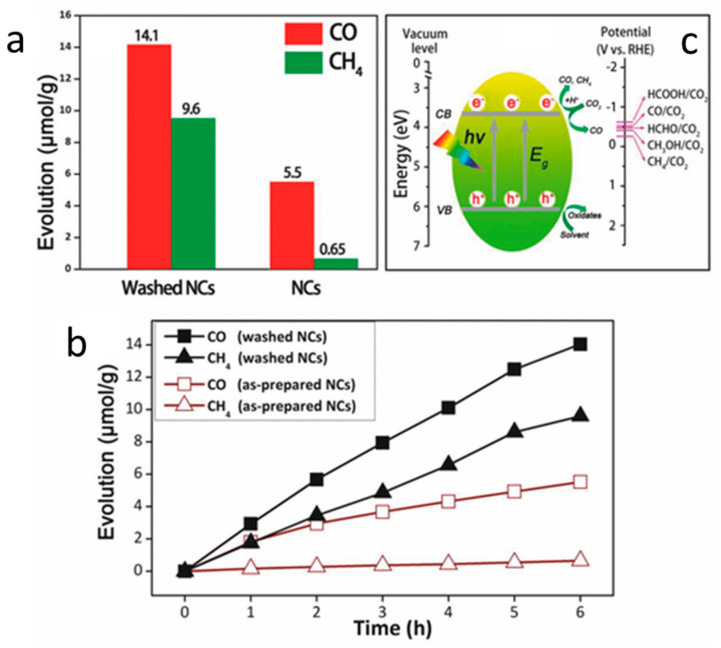
(**a**) CO and CH_4_ formation, (**b**) times course product formation in different samples of Cs_2_AgBiBr_6_, and (**c**) Schematic illustration of CO_2_ reduction on the surface of Cs_2_AgBiBr_6_. Reproduced from [104], with permission from John Wiley and Sons, 2018.

Recently, halide double perovskite materials have been recognized as the ideal alternative for toxic lead halide perovskites [98]. Numerous experiments have demonstrated excellent optoelectronic features of halide double perovskites, which are also suitable for photocatalytic CO_2_ applications [102,103]. In 2018, Zhou et al. demonstrated a highly crystalline Cs_2_AgBiBr_6_ double perovskite NCs synthesized through a hot injection process [104]. To acquire the highly crystalline Cs_2_AgBiBr_6_, the temperature was optimized, and it was observed that 200 °C is a suitable temperature to get a pure form of double perovskite. The significant role of OLA and OA ligands towards the formation of crystalline Cs_2_AgBiBr_6_ was studied, where it was observed that in the absence of these ligands, bulk Cs_2_AgBiBr_6_ was formed. The bandgap was calculated to be 2.52 eV by Tauc’s plot, and band potentials were measured by combining the results of VB-XPS and bandgap values. The enlarged band gap was observed in NCs as compared to bulk Cs_2_AgBiBr_6_, which was attributed to its quantum confinement effect. The stability of the NCs was studied in different solvents ranging from polar, partial polar, non-polar, and protonic solvents. Results revealed that the NCs were quickly decomposed in polar solvents like DMF or acetone and highly stable in mild/non-polar solvents for 3 weeks. The high ligand density on the surface of NCs may block electron/hole transportation and decrease the catalytic activity. Therefore, to decline the ligand density, the NCs were washed with absolute ethanol, which was supposed to improve the catalytic activity. The XPS and FTIR revealed that the surface ligands were wholly removed by the washing process (Figure 8a, b), while TGA results further confirm the removal of organic residues (Figure 8c, d). As shown in Figure 9a, b, the catalytic activity of CO_2_ reduction towards CO and CH_4_ evolution was much higher in the NCs washed by absolute ethanol than the Cs_2_AgBiBr_6_ NCs without the washed one in 6 h. The band potentials of the NCs are well suitable for the reduction of CO_2_ to produce CO/CH_4_, as shown in Figure 9c. The stability of catalysts was examined by post catalytic analysis using TEM, XRD, and XPS analysis. The results revealed that the catalyst’s structure and the surface did not differ from those of fresh samples. The further extension for the development of the Z-scheme Cs_2_AgBiBr_6_@g-C_3_N_4_ was carried out by Wang and coworkers [105]. 

**Figure 10:**
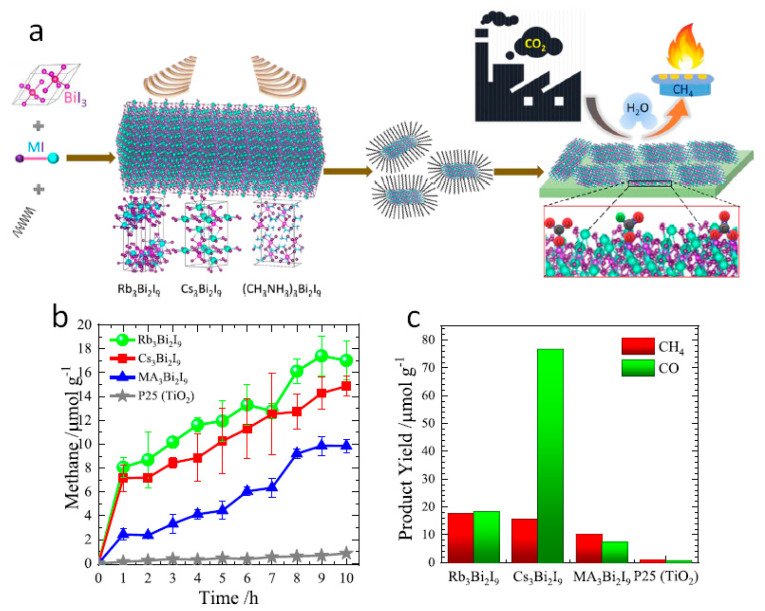
(**a**) Schematic illustration of the synthesis of Bi−based perovskites by top-down method, (**b**) Time−dependent CH_4_ production in Bi−based perovskites, and (**c**) yields of CO and CH_4_ production in different samples. Reproduced from [107], with permission from American Chemical Society, 2019.

The Z-scheme combination was achieved by the in-situ method, mixing g-C_3_N_4_ precursor to Cs_2_AgBiBr_6_ nanoparticles in dichloromethane/toluene. The optimized catalyst achieved 2.0 µmol g^−1^ h^−1^ of activity for CO and CH_4_ production with CH_4_ selectivity over 70%. The construction of Z-scheme among perovskite and g-C_3_N_4_ improves the redox ability of the system. After that, Sn-based halide perovskites fell into the spotlight as Pb-free materials. Wang et al. successfully developed the novel Cs_2_SnI_6_/SnS_2_ nanosheet combination in 2019 [106]. Such a heterojunction mixture of perovskite NCs with metal dichalcogenide (SnS_2_) nanosheets greatly increases the lifetime of photogenerated electrons from 1290 to 3080 ps, observed from transient absorption measurements. DFT studies confirmed the type-II band alignment in Cs_2_SnI_6_/SnS_2_ heterojunction, which was further supported by UPS measurement. Such a heterojunction was responsible for improved electron transportation through Cs_2_SnI_6_ and SnS_2_ interface, and hole extraction by Cs_2_SnI_6_ from SnS_2_, defeating the electron-hole recombination. As a result, the 5.4 times improved activity was observed in the Cs_2_SnI_6_(1.0)/SnS_2_ sample (CH_4_, 6.09 µmol g^−1^) compared to the pristine SnS_2_ and the stability of 3 cycles. No changes in the XRD pattern and UV-Vis-NIR spectra were observed in the samples tested after CO_2_ reduction. Apart from Sn-based perovskites, Bi-based materials are considered the best replacement for Pb-materials [107]. In this regard, Bhosale et al. developed a system anchored series of non-toxic, Bi-based halide perovskites, such as Rb_3_Bi_2_I_9_, Cs_3_Bi_2_I_9_, and MA_3_Bi_2_I_9_ by an ultrasonic, top-down method (Figure 10a). The catalyst showed 12 h of stability after seven days of aging under UV illumination, confirmed by XRD patterns. The catalytic responses were acquired at a gas-solid interface under UV irradiation. The time-dependent CH_4_ evolution was measured for 10 h illumination, and increased CH_4_ production was observed in all the samples (Figure 10b). The comparative catalyst activity for Bi-based perovskite was observed in the order of Cs_3_Bi_2_I_9_ > Rb_3_Bi_2_I_9_ > MA_3_Bi_2_I_9_ towards CO and Rb_3_Bi_2_I_9_ > Cs_3_Bi_2_I_9_ > MA_3_Bi_2_I_9_ for CH_4_ evolution after 10 h UV illumination (Figure 10c).

Later, halide perovskite confined with Sb metal center (i.e., Cs_3_Sb_2_Br_9_) was developed by Lu et al. and showed 10 times better activity than CsPbBr_3_ NCs [108]. The effect of the ligand in the hot injection synthesis of Cs_3_Sb_2_Br_9_ from CsPbX_3_ was studied. It was revealed that the use of saturated octanoic acid by replacing unsaturated oleic acid produces pure Cs_3_Sb_2_Br_9_ NCs due to the temperature expansion up to 230 °C. Moreover, these ligands were purified/removed by simple hexane/acetone washings before CO_2_ reduction tests. Resulting, the photocatalytic CO_2_ reduction of Cs_3_Sb_2_Br_9_ was carried out in the presence of dried octadecene solvent. The octadecene plays an important role; (i) it has low volatility and (ii) increases the solubility of CO_2_ compared to typical solvents like acetonitrile or ethyl acetate. After 4 h light illumination, Cs_3_Sb_2_Br_9_ generated 510 µmol g^−1^ of CO, which was over 10 times greater than various halide perovskites. The DFT calculations revealed that the sites Cs_3_Sb_2_Br_9_ on the (1000) and (0001) surfaces play an essential role in forming COOH* and CO* intermediates. Hence, such studies may help establish a practical, large-scale, Pb-free photocatalyst for the CO_2_ reduction process in the near future. The summary of all the studied catalysts has been presented in Table 2.

## 5. Summary and Outlook

Halide perovskites have been considered an advanced and high-performance material for many applications over the last few years. Their superior optoelectronic properties make them suitable for photocatalytic CO_2_ reduction. Pb-halide perovskites have proven to be the finest materials for CO_2_ conversion due to their high catalytic activity, high stability towards humidity, and long-term photostability. However, due to Pb’s high toxicity, the research focus is shifting towards the development of non-toxic, Pb-free halide perovskites. Therefore, in this review, the Pb and Pb-free halide perovskite’s recent progress towards photocatalytic CO_2_ reduction has been involved. We have covered the halide perovskites and their hybrid heterostructures/composites formed with metal co-catalyst, graphene, metal complexes, MOFs, and other 0D or 2D semiconductors. Although halide perovskites achieved significant success for photoreduction of CO_2_; still the catalytic efficiency is restricted in the µmol range, which keeps them away from large-scale use. Almost all the reported halide perovskites undergo CO_2_ reduction to form C_1_ products like CO and CH_4_. Therefore, more focus should be given to generate higher hydrocarbons, which are industrially important. Therefore, understanding the reaction mechanism towards the formation of higher-ordered organic chemicals via CO_2_ reduction of perovskite structure is crucial. Moreover, it is necessary to emphasize improving the structural and chemical stability of these materials. Improved catalytic efficiency and stability can be achieved by combining halide perovskite with other efficient semiconductors, improving the optical behavior, and charge separation ability. Therefore, developing sustainable, scalable, and low-cost halide perovskites will be a tremendous challenge for real applications. We believe that the process of unlocking more perovskite materials with improved optoelectronic features should continue to make them perfect for better CO_2_ reduction performance.

## Figures and Tables

**Table 1:** The catalytic pathway of CO_2_ reduction towards various main products with their redox potentials (at pH = 7).

No.	Reaction	*E*^0^_redox Vs_. _NHE_	Product
1	CO_2_ + e^−^ → CO_2_^●−^	−1.90 V	CO_2_^●−^
2	CO_2_ + 2H^+^ + 2e^−^ → CO + H_2_O	−0.53 V	Carbon monoxide
3	CO_2_ + 2H^+^ + 2e^−^ → HCOOH	−0.61 V	Formic acid
4	CO_2_ + 4H^+^ + 4e^−^ → HCHO + H_2_O	−0.48 V	Formaldehyde
5	CO_2_ + 6H^+^ + 6e^−^ → CH_3_OH + H_2_O	−0.38 V	Methanol
6	CO_2_ + 8H^+^ + 8e^−^ → CH_4_ + 2H_2_O	−0.24 V	Methane
7	2CO_2_ + 8H^+^ + 8e^−^ → CH_3_COOH + 2H_2_O	−0.31 V	Acetic acid
8	2CO_2_ + 14H^+^ + 14e^−^ → C_2_H_6_ + 4H_2_O	−0.51 V	Ethane

**Table 2:** The progress of Pb and Pb-free perovskites for CO_2_ reduction with details including catalysts [reference], reaction medium, light source, product yield and time, and stability (*EA = ethyl acetate, MeOH = methanol, AN = acetonitrile, IPN = isopropanol, TCM = trichloromethane, OC = octadecene).

No	Catalyst	Medium	Light Source	Product, Yield, and Reaction Time	Catalytic Stability
Lead-based halide perovskites					
1	Fe/CH_3_NH_3_PbI_3_ (MAPbI_3_) QDs [60]	EA/H_2_O	300 W Xe-lamp with standard 400 nm filter	CO + CH_4_, 1559 µmol g^−1^ CO (34%) and CH_4_ (66%)	80 h
2	CsPbBr_3_ QDs [61]	EA/H_2_O	300 W Xe-lamp with standard AM 1.5 filter	CO, 20.9 µmol g^−1^ (average electron yield)	8 h
3	CsPb(Br_0.5_/Cl_0.5_)_3_ [62]	EA	300 W Xe-lamp with AM 1.5 filter	CO, 767 µmol g^−1^ (9 h) CH_4_, 108 µmol g^−1^ (9 h)	9 h
4	Co- and Fe-CsPbBr_3_ [63]	A theoretical study, DFT calculations with DMol_3_ program			
5	Fe(II)-CsPbBr_3_ [64]	EA/H_2_O	300 W Xe-lamp (150 mW cm^−2^ light intensity)	CO, 6.1 µmol g^−1^ h^−1^ (3 h) CH_4_, 3.2 µmol g^−1^ h^−1^ (3 h)	-
6	Mn/CsPb(Br/Cl)_3_ [65]	EA	300 W Xe-lamp with AM 1.5 filter	CO, 1917 µmol g^−1^ (9 h) CH_4_, 82 µmol g^−1^ (9 h)	9 h
7	Co-CsPbBr_3_/Cs_4_PbBr_6_ [59]	H_2_O	Xe-lamp irradiation with a 400 nm filter (100 mW cm^−2^ light intensity)	CO, 239 µmol g^−1^ (20 h) CH_4_, 7 µmol g^−1^ (20 h)	-
8	Co-CsPbBr_3_/Cs_4_PbBr_6_ [66]	AN/H_2_O/MeOH	300 W Xe-lamp (light intensity of 100 mW m^−2^)	CO, 1835 µmol g^−1^ (15 h)	-
9	Pt/CsPbBr_3_ [67]	EA	150 W Xe-lamp with 380 nm cut off filter	CO, 5.6 µmol g^−1^ h^−1^	30 h
10	Ni and Mn-doped CsPbCl_3_ NCs [68]	CO_2_/ H_2_O	300 W Xe-lamp with AM 1.5 filter	Ni = CO, 169.37 μmol g^−1^ h^−1^ Mn = 152.49 μmol g^−1^ h^−1^	6 h (3 runs)
11	CsPbBr_3_ QDs/GO [71]	EA	100 W Xe-lamp with AM 1.5 filter	CO, 58.7 µmol g^−1^ (12 h) CH_4_, 29.6 µmol g^−1^ (12 h) H_2_, 1.58 µmol g^−1^ (12 h)	12 h
12	Cs_4_PbBr_6_/rGO [73]	EA/H_2_O	300 W Xe-lamp with 420 nm filter (light intensity, 100 mW cm^−2^)	CO, 11.4 μmol g^−1^ h^−1^	60 h
13	Cu-RGO-CsPbBr_3_ [72]	CO_2_/H_2_O	Xe-lamp irradiation with a 400 nm filter	CH_4_ 12.7 μmol g^–1^ h^–1^ (4 h)	12 h (3 cycles)
14	CsPbBr_3_/USGO/α-Fe_2_O_3_ [74]	ACN/H_2_O	300 W Xe-lamp with 420 nm filter (light intensity, 100 mW cm^−2^)	CO, 73.8 μmol g^−1^ h^−1^	2 cycles
15	CsPbBr_3_/Bi_2_WO_6_ [75]		300 W Xe-lamp with 420 nm filter (100 mW cm^−2^ light intensity)	CH_4_/CO, 503 μmol g^–1^	4 Runs (pristine samples)
16	CsPbBr_3_/Pd Nanosheet [76]	H_2_O vapor	A 150 W Xe-lamp (Zolix) equipped with an AM 1.5 G and 420 nm optical filter (100 mW cm^−2^ light intensity)	CO, 12.63 µmol g^−1^ (3 h) CH_4_, 10.41 µmol g^−1^ (3 h) Electron consumption rate, 101.39 µmol g^−1^ (3 h)	-
17	CsPbBr_3_/MXene Nanosheets [77]	EA	300 W Xe-lamp with 420 nm cut-off filter	CO, 26.32 µmol g^−1^ h^−1^ CH_4_, 7.25 µmol g^−1^ h^−1^	-
18	Amorphous-TiO_2_/CsPbBr_3_ NCs [78]	EA/IPN	150 W Xe-lamp with an AM 1.5 G filter	CO, 11.71 μmol g ^−1^ CH_4_, 20.15 μmol g ^−1^ H_2_, 4.38 μmol g ^−1^	30 h
19	TiO_2_/CsPbBr_3_ [79]	ACN/H_2_O	300 W Xe-arc lamp	CO, 9.02 μmol g^–1^ h^–1^	16 h
20	CsPbBr_3_ NCs- ZnO nanowire/graphene [81]	CO_2_/H_2_O	A 150 W Xe-lamp with an AM 1.5 G and 420 nm optical filter (100 mW cm^−2^ light intensity)	CH_4_, 6.29 µmol g^−1^ h^−1^ (3 h) CO, 0.8 µmol g^−1^ h^−1^ (3 h) Photoelectron consumption rate, 52.02 µmol g^−1^ h^−1^ (3 h)	4 cycles
21	CsPbBr_3_ QDs/g-C_3_N_4_ [83]	ACN/H_2_O	300 W Xe-lamp with a 420 nm cut-off filter	CO, 149 μmol g^−1^ h^−1^	3 Runs
22	CsPbBr_3_/g-C_3_N_4_ containing TiO species [84]	EA/H_2_O	Xe-lamp with a 400 nm cut off filter (100 mW cm^−2^ light intensity)	CO, 129 μmol g^−1^ (10 h)	-
23	CsPbBr_3_@Zeolitic Imidazolate [85]	CO_2_/ H_2_O	100 W Xe-lamp with AM 1.5 G filter (light intensity was 150 mW cm^−2^)	The electron consumption rate for CH_4_, 29.630 μmol g^–1^ h^–1^ (3 h)	6 cycles
24	CsPbBr_3_ QDs/UiO-66(NH_2_) nanojunction [86]	EA/H_2_O	300 W Xe-lamp with a 420 nm UV-cut filter	CO, 98.57 μmo g^−1^ CH_4_, 3.08 μmol g^−1^ (12 h)	3 cycles
25	[Ni(terpy)_2_]^2+^ (Ni(tpy)) CsPbBr_3_ NCs [93]	EA/H_2_O	300 W Xe-lamp (Solaredge 700, 100 mW cm^−2^), λ > 400 nm	CO + CH_4_, 1724 μmol g^−1 ^ (4 h)	16 h
26	CsPbBr_3_-Re(CO)_3_Br(dcbpy) (dcbpy¼4,4′- dicarboxy-2,2′-bipyridine) [92]	Toluene/IPN	150 W Xe-lamp (≥420 nm)	CO, 509.14 μmol g^−1^ (15 h)	-
27	FAPbBr_3_ QDs [94]	EA/H_2_O	300 W Xe-lamp (light intensity of 100 mW cm^−2^)	CO, 181.25 μmol g^−1^ h^−1^	-
Lead-free halide perovskites					
28	Cs_2_AgBiBr_6_ [104]	EA	100 W Xe-lamp with an AM 1.5 G filter	CO, 14.1 μmol g^−1^ (6 h) CH_4_, 9.6 μmol g^−1^ (6 h)	6 h
29	Cs_2_AgBiBr_6_@g-C_3_N_4_ Z-scheme [105]	EA/MeOH	Xe-lamp (80 mW cm^−2^ light intensity)	CO + CH_4_, 2.0 μmol g^−1^ h^−1^	12 h
30	Cs_2_SnI_6_/SnS_2_ Nanosheet [106]	CO_2_/H_2_O/MeOH	150 mW cm^−2^ Xe-lamp with 400 nm filter	CH_4_, 6.09 μmol g^−1^ (3 h)	9 h (3 cycles)
31	Bi-based perovskite NCs [107] Rb_3_Bi_2_I_2_ Cs_3_Bi_2_I_9_ MA_3_Bi_2_T_9_	TCM	32 W UV-lamp, 305 nm	Cs_3_Bi_2_I_9_ = CO, 77.6 μmol g^−1^ and CH_4_, 14.9 μmol g^−1^ (10 h) Rb_3_Bi_2_I_9_ = CO, 18.2 μmol g^−1^ and CH_4_, 17.0 μmol g^−1^ (10 h) MA_3_Bi_2_I_9_ = CO, 7.2 μmol g^−1^ and CH_4_, 9.8 μmol g^−1^ (10 h)	-
32	Cs_3_Sb_2_Br_9_ [108]	Dried OC	300 W Xe-lamp with AM 1.5 irradiation	CO, 510 μmol g^−1^ (4 h)	9 h

## References

[1] Li X., Yu J., Jaroniec M., Chen X. (2019). Cocatalysts for selective photoreduction of CO_2_ into solar fuels. Chem. Rev..

[2] Park S.-M., Razzaq A., Park Y.H., Sorcar S., Park Y., Grimes C.A., In S.-I. (2016). Hybrid Cu_x_O–TiO_2_ Heterostructured Composites for Photocatalytic CO_2_ Reduction into Methane Using Solar Irradiation: Sunlight into Fuel. ACS Omega.

[3] Kim K., Razzaq A., Sorcar S., Park Y., Grimes C.A., In S.-I. (2016). Hybrid mesoporous Cu_2_ZnSnS_4_(CZTS)–TiO_2_ photocatalyst for efficient photocatalytic conversion of CO_2_ into CH_4_ under solar irradiation. RSC Adv..

[4] Parayil S.K., Razzaq A., Park S.-M., Kim H.R., Grimes C.A., In S.-I. (2015). Photocatalytic conversion of CO_2_ to hydrocarbon fuel using carbon and nitrogen co-doped sodium titanate nanotubes. Appl. Catal. A Gen..

[5] Kim H.R., Razzaq A., Grimes C.A., In S.-I. (2017). Heterojunction pnp Cu_2_O/S-TiO_2_/CuO: Synthesis and application to photocatalytic conversion of CO_2_ to methane. J. CO2 Util..

[6] Sorcar S., Hwang Y., Lee J., Kim H., Grimes K.M., Grimes C.A., Jung J.-W., Cho C.-H., Majima T., Hoffmann M.R. (2019). CO_2_, water, and sunlight to hydrocarbon fuels: A sustained sunlight to fuel (Joule-to-Joule) photoconversion efficiency of 1%. Energy Environ. Sci..

[7] Ali S., Flores M.C., Razzaq A., Sorcar S., Hiragond C.B., Kim H.R., Park Y.H., Hwang Y., Kim H.S., Kim H. (2019). Gas Phase Photocatalytic CO_2_ Reduction,“A Brief Overview for Benchmarking”. Catalysts.

[8] Hiragond C., Ali S., Sorcar S., In S.-I. (2019). Hierarchical Nanostructured Photocatalysts for CO_2_ Photoreduction. Catalysts.

[9] Sorcar S., Thompson J., Hwang Y., Park Y.H., Majima T., Grimes C.A., Durrant J.R., In S.-I. (2018). High-rate solar-light photoconversion of CO_2_ to fuel: Controllable transformation from C_1_ to C_2_ products. Energy Environ. Sci..

[10] Sorcar S., Hwang Y., Grimes C.A., In S.-I. (2017). Highly enhanced and stable activity of defect-induced titania nanoparticles for solar light-driven CO_2_ reduction into CH_4_. Mater. Today.

[11] Ali S., Lee J., Kim H., Hwang Y., Razzaq A., Jung J.-W., Cho C.-H., In S.-I. (2020). Sustained, Photocatalytic CO_2_ Reduction to CH_4_ in a Continuous Flow Reactor by Earth-Abundant Materials: Reduced Titania-Cu_2_O Z-Scheme Heterostructures. Appl. Catal. B Environ..

[12] Kumar A., Thakur P.R., Sharma G., Naushad M., Rana A., Mola G.T., Stadler F.J. (2019). Carbon nitride, metal nitrides, phosphides, chalcogenides, perovskites and carbides nanophotocatalysts for environmental applications. Environ. Chem. Lett..

[13] Das S., Daud W.M.A.W. (2014). A review on advances in photocatalysts towards CO_2_ conversion. Rsc Adv..

[14] Razzaq A., Sinhamahapatra A., Kang T.-H., Grimes C.A., Yu J.-S., In S.-I. (2017). Efficient solar light photoreduction of CO_2_ to hydrocarbon fuels via magnesiothermally reduced TiO_2_ photocatalyst. Appl. Catal. B Environ..

[15] Akhundi A., Habibi-Yangjeh A., Abitorabi M., Rahim Pouran S. (2019). Review on photocatalytic conversion of carbon dioxide to value-added compounds and renewable fuels by graphitic carbon nitride-based photocatalysts. Catal. Rev..

[16] Wu H., Li X., Tung C., Wu L. (2019). Semiconductor Quantum Dots: An Emerging Candidate for CO_2_ Photoreduction. Adv. Mater..

[17] Jiao X., Zheng K., Liang L., Li X., Sun Y., Xie Y. (2020). Fundamentals and challenges of ultrathin 2D photocatalysts in boosting CO_2_ photoreduction. Chem. Soc. Rev..

[18] Nguyen T.P., Nguyen D.L.T., Nguyen V.-H., Le T.-H., Vo D.-V.N., Trinh Q.T., Bae S.-R., Chae S.Y., Kim S.Y., Le Q. (2020). Van Recent Advances in TiO_2_-Based Photocatalysts for Reduction of CO_2_ to Fuels. Nanomaterials.

[19] Kim M., Razzaq A., Kim Y.K., Kim S., In S.-I. (2014). Synthesis and characterization of platinum modified TiO_2_-embedded carbon nanofibers for solar hydrogen generation. RSC Adv..

[20] Razzaq A., In S.-I. (2019). TiO_2_ Based Nanostructures for Photocatalytic CO_2_ Conversion to Valuable Chemicals. Micromachines.

[21] Zubair M., Razzaq A., Grimes C.A., In S.-I. (2017). Cu_2_ZnSnS_4_ (CZTS)-ZnO: A noble metal-free hybrid Z-scheme photocatalyst for enhanced solar-spectrum photocatalytic conversion of CO_2_ to CH_4_. J. CO2 Util..

[22] Razzaq A., Ali S., Asif M., In S.-I. (2020). Layered Double Hydroxide (LDH) Based Photocatalysts: An Outstanding Strategy for Efficient Photocatalytic CO_2_ Conversion. Catalysts.

[23] Hiragond C.B., Lee J., Kim H., Jung J.-W., Cho C.-H., In S.-I. (2020). A novel N-doped graphene oxide enfolded reduced titania for highly stable and selective gas-phase photocatalytic CO_2_ reduction into CH_4_: An in-depth study on the interfacial charge transfer mechanism. Chem. Eng. J..

[24] Zeng S., Kar P., Thakur U.K., Shankar K. (2018). A review on photocatalytic CO_2_ reduction using perovskite oxide nanomaterials. Nanotechnology.

[25] Jena A.K., Kulkarni A., Miyasaka T. (2019). Halide perovskite photovoltaics: Background, status, and future prospects. Chem. Rev..

[26] Johnsson M., Lemmens P. (2007). Crystallography and chemistry of perovskites. Handb. Magn. Adv. Magn. Mater..

[27] Xue J., Wang R., Yang Y. (2020). The surface of halide perovskites from nano to bulk. Nat. Rev. Mater..

[28] Teh Y.W., Chee M.K.T., Kong X.Y., Yong S.-T., Chai S.-P. (2020). An insight into perovskite-based photocatalysts for artificial photosynthesis. Sustain. Energy Fuels.

[29] Hemminger J.C., Carr R., Somorjai G.A. (1978). The photoassisted reaction of gaseous water and carbon dioxide adsorbed on the SrTiO_3_ (111) crystal face to form methane. Chem. Phys. Lett..

[30] Luo C., Zhao J., Li Y., Zhao W., Zeng Y., Wang C. (2018). Photocatalytic CO_2_ reduction over SrTiO_3_: Correlation between surface structure and activity. Appl. Surf. Sci..

[31] Kwak B.S., Do J.Y., Park N.-K., Kang M. (2017). Surface modification of layered perovskite Sr_2_TiO_4_ for improved CO_2_ photoreduction with H_2_O to CH_4_. Sci. Rep..

[32] Li P., Ouyang S., Xi G., Kako T., Ye J. (2012). The effects of crystal structure and electronic structure on photocatalytic H_2_ evolution and CO_2_ reduction over two phases of perovskite-structured NaNbO_3_. J. Phys. Chem. C.

[33] Wang Y., Liu M., Chen W., Mao L., Shangguan W. (2019). Ag loaded on layered perovskite H_2_SrTa_2_O_7_ to enhance the selectivity of photocatalytic CO_2_ reduction with H_2_O. J. Alloys Compd..

[34] Fan J., Jia B., Gu M. (2014). Perovskite-based low-cost and high-efficiency hybrid halide solar cells. Photonics Res..

[35] Kulkarni S.A., Mhaisalkar S.G., Mathews N., Boix P.P. (2019). Perovskite nanoparticles: Synthesis, properties, and novel applications in photovoltaics and LEDs. Small Methods.

[36] Kubicek M., Bork A.H., Rupp J.L.M. (2017). Perovskite oxides–a review on a versatile material class for solar-to-fuel conversion processes. J. Mater. Chem. A.

[37] Chen P., Ong W., Shi Z., Zhao X., Li N. (2020). Pb-Based Halide Perovskites: Recent Advances in Photo (electro) catalytic Applications and Looking Beyond. Adv. Funct. Mater..

[38] Tabish A., Varghese A.M., Wahab M.A., Karanikolos G.N. (2020). Perovskites in the Energy Grid and CO_2_ Conversion: Current Context and Future Directions. Catalysts.

[39] Singh M., Sinha I. (2020). Halide perovskite-based photocatalysis systems for solar-driven fuel generation. Sol. Energy.

[40] Shyamal S., Pradhan N. (2020). Halide Perovskite Nanocrystal Photocatalysts for CO_2_ Reduction: Successes and Challenges. J. Phys. Chem. Lett..

[41] Han C., Zhu X., Martin J.S., Lin Y., Spears S., Yan Y. (2020). Recent Progress in Engineering Metal Halide Perovskites for Efficient Visible-Light-Driven Photocatalysis. ChemSusChem.

[42] Huang H., Pradhan B., Hofkens J., Roeffaers M.B.J., Steele J.A. (2020). Solar-Driven Metal Halide Perovskite Photocatalysis: Design, Stability, and Performance. ACS Energy Lett..

[43] Li P., Liu L., An W., Wang H., Guo H., Liang Y., Cui W. (2020). Ultrathin porous g-C_3_N_4_ nanosheets modified with AuCu alloy nanoparticles and C-C coupling photothermal catalytic reduction of CO_2_ to ethanol. Appl. Catal. B Environ..

[44] Li K., Peng B., Peng T. (2016). Recent advances in heterogeneous photocatalytic CO_2_ conversion to solar fuels. ACS Catal..

[45] Lingampalli S.R., Ayyub M.M., Rao C.N.R. (2017). Recent progress in the photocatalytic reduction of carbon dioxide. ACS Omega.

[46] Qin D., Zhou Y., Wang W., Zhang C., Zeng G., Huang D., Wang L., Wang H., Yang Y., Lei L. (2020). Recent advances in two-dimensional nanomaterials for photocatalytic reduction of CO_2_: Insights into performance, theories and perspective. J. Mater. Chem. A.

[47] Thompson W.A., Sanchez Fernandez E., Maroto-Valer M.M. (2020). Review and Analysis of CO_2_ Photoreduction Kinetics. ACS Sustain. Chem. Eng..

[48] Chang X., Wang T., Gong J. (2016). CO 2 photo-reduction: Insights into CO 2 activation and reaction on surfaces of photocatalysts. Energy Environ. Sci..

[49] Peng C., Reid G., Wang H., Hu P. (2017). Perspective: Photocatalytic reduction of CO_2_ to solar fuels over semiconductors. J. Chem. Phys..

[50] Zhang Y., Mori T., Ye J. (2012). Polymeric carbon nitrides: Semiconducting properties and emerging applications in photocatalysis and photoelectrochemical energy conversion. Sci. Adv. Mater..

[51] Zhu X., Lin Y., Sun Y., Beard M.C., Yan Y. (2019). Lead-halide perovskites for photocatalytic α-alkylation of aldehydes. J. Am. Chem. Soc..

[52] Kishan K.Y., Bisht N., Hiragond C., Dey A., Khanna P.K., More P. (2020). V Room temperature thermoelectric performance of Methyl Ammonium Lead Iodide Perovskite and their MWCNT-PANI composites. Mater. Today Chem..

[53] Zhu X., Lin Y., San Martin J., Sun Y., Zhu D., Yan Y. (2019). Lead halide perovskites for photocatalytic organic synthesis. Nat. Commun..

[54] Yang Y., Yang M., Li Z., Crisp R., Zhu K., Beard M.C. (2015). Comparison of recombination dynamics in CH_3_NH_3_PbBr_3_ and CH_3_NH_3_PbI_3_ perovskite films: Influence of exciton binding energy. J. Phys. Chem. Lett..

[55] Wu Y., Wang P., Zhu X., Zhang Q., Wang Z., Liu Y., Zou G., Dai Y., Whangbo M., Huang B. (2018). Composite of CH_3_NH_3_PbI_3_ with reduced graphene oxide as a highly efficient and stable visible-light photocatalyst for hydrogen evolution in aqueous HI solution. Adv. Mater..

[56] Wu Y., Wang P., Guan Z., Liu J., Wang Z., Zheng Z., Jin S., Dai Y., Whangbo M.-H., Huang B. (2018). Enhancing the Photocatalytic Hydrogen Evolution Activity of Mixed-Halide Perovskite CH_3_NH_3_PbBr_3–x_I_x_ Achieved by Bandgap Funneling of Charge Carriers. ACS Catal..

[57] Cardenas-Morcoso D., Gualdrón-Reyes A.F., Ferreira Vitoreti A.B., García-Tecedor M., Yoon S.J., Solis de la Fuente M., Mora-Seró I., Gimenez S. (2019). Photocatalytic and Photoelectrochemical Degradation of Organic Compounds with All-Inorganic Metal Halide Perovskite Quantum Dots. J. Phys. Chem. Lett..

[58] Gao G., Xi Q., Zhou H., Zhao Y., Wu C., Wang L., Guo P., Xu J. (2017). Novel inorganic perovskite quantum dots for photocatalysis. Nanoscale.

[59] Mu Y., Zhang W., Guo X., Dong G., Zhang M., Lu T. (2019). Water-Tolerant Lead Halide Perovskite Nanocrystals as Efficient Photocatalysts for Visible-Light-Driven CO_2_ Reduction in Pure Water. ChemSusChem.

[60] Wu L., Mu Y., Guo X., Zhang W., Zhang Z., Zhang M., Lu T. (2019). Encapsulating Perovskite Quantum Dots in Iron-Based Metal–Organic Frameworks (MOFs) for Efficient Photocatalytic CO_2_ Reduction. Angew. Chemie Int. Ed..

[61] Hou J., Cao S., Wu Y., Gao Z., Liang F., Sun Y., Lin Z., Sun L. (2017). Inorganic colloidal perovskite quantum dots for robust solar CO_2_ reduction. Chem. Eur. J..

[62] Guo S.-H., Zhou J., Zhao X., Sun C.-Y., You S.-Q., Wang X.-L., Su Z.-M. (2019). Enhanced CO_2_ photoreduction via tuning halides in perovskites. J. Catal..

[63] Tang C., Chen C., Xu W., Xu L. (2019). Design of doped cesium lead halide perovskite as a photo-catalytic CO_2_ reduction catalyst. J. Mater. Chem. A.

[64] Shyamal S., Dutta S.K., Pradhan N. (2019). Doping Iron in CsPbBr_3_ Perovskite Nanocrystals for Efficient and Product Selective CO_2_ Reduction. J. Phys. Chem. Lett..

[65] Liu Y.-W., Guo S.-H., You S.-Q., Sun C.-Y., Wang X.-L., Zhao L., Su Z.-M. (2020). Mn-doped CsPb(Br/Cl)_3_ mixed-halide perovskites for CO_2_ photoreduction. Nanotechnology.

[66] Dong G.-X., Zhang W., Mu Y.-F., Su K., Zhang M., Lu T.-B. (2020). A halide perovskite as a catalyst to simultaneously achieve efficient photocatalytic CO_2_ reduction and methanol oxidation. Chem. Commun..

[67] Chen Y.-X., Xu Y.-F., Wang X.-D., Chen H.-Y., Kuang D.-B. (2020). Solvent selection and Pt decoration towards enhanced photocatalytic CO_2_ reduction over CsPbBr_3_ perovskite single crystals. Sustain. Energy Fuels.

[68] Zhu J., Zhu Y., Huang J., Hou L., Shen J., Li C. (2020). Synthesis of monodisperse water-stable surface Pb-rich CsPbCl_3_ nanocrystals for efficient photocatalytic CO_2_ reduction. Nanoscale.

[69] Hiragond C.B., Kim H., Lee J., Sorcar S., Erkey C., In S.-I. (2020). Electrochemical CO_2_ Reduction to CO Catalyzed by 2D Nanostructures. Catalysts.

[70] Ali S., Razzaq A., In S.-I. (2019). Development of graphene based photocatalysts for CO_2_ reduction to C_1_ chemicals: A brief overview. Cat. Today.

[71] Xu Y.-F., Yang M.-Z., Chen B.-X., Wang X.-D., Chen H.-Y., Kuang D.-B., Su C.-Y. (2017). A CsPbBr_3_ perovskite quantum dot/graphene oxide composite for photocatalytic CO_2_ reduction. J. Am. Chem. Soc..

[72] Kumar S., Regue M., Isaacs M.A., Freeman E., Eslava S. (2020). All-Inorganic CsPbBr_3_ Nanocrystals: Gram-Scale Mechanochemical Synthesis and Selective Photocatalytic CO_2_ Reduction to Methane. ACS Appl. Energy Mater..

[73] Wang X., Li K., He J., Yang J., Dong F., Mai W., Zhu M. (2020). Defect in reduced graphene oxide tailored selectivity of photocatalytic CO_2_ reduction on Cs_4_PbBr_6_ pervoskite hole-in-microdisk structure. Nano Energy.

[74] Mu Y., Zhang W., Dong G., Su K., Zhang M., Lu T. (2020). Ultrathin and Small-Size Graphene Oxide as an Electron Mediator for Perovskite-Based Z-Scheme System to Significantly Enhance Photocatalytic CO_2_ Reduction. Small.

[75] Wang J., Wang J., Li N., Du X., Ma J., He C., Li Z. (2020). Direct Z-Scheme 0D/2D Heterojunction of CsPbBr_3_ Quantum Dots/Bi_2_WO_6_ Nanosheets for Efficient Photocatalytic CO_2_ Reduction. ACS Appl. Mater. Interfaces.

[76] Xu Y.-F., Yang M.-Z., Chen H.-Y., Liao J.-F., Wang X.-D., Kuang D.-B. (2018). Enhanced solar-driven gaseous CO_2_ conversion by CsPbBr_3_ nanocrystal/Pd nanosheet Schottky-junction photocatalyst. ACS Appl. Energy Mater..

[77] Pan A., Ma X., Huang S., Wu Y., Jia M., Shi Y., Liu Y., Wangyang P., He L., Liu Y. (2019). CsPbBr_3_ Perovskite Nanocrystal Grown on MXene Nanosheets for Enhanced Photoelectric Detection and Photocatalytic CO_2_ Reduction. J. Phys. Chem. Lett..

[78] Xu Y., Wang X., Liao J., Chen B., Chen H., Kuang D. (2018). Amorphous-TiO_2_-Encapsulated CsPbBr_3_ Nanocrystal Composite Photocatalyst with Enhanced Charge Separation and CO_2_ Fixation. Adv. Mater. Interfaces.

[79] Xu F., Meng K., Cheng B., Wang S., Xu J., Yu J. (2020). Unique S-scheme heterojunctions in self-assembled TiO_2_/CsPbBr_3_ hybrids for CO_2_ photoreduction. Nat. Commun..

[80] Lu Y., Ma Y., Zhang T., Yang Y., Wei L., Chen Y. (2018). Monolithic 3D cross-linked polymeric graphene materials and the likes: Preparation and their redox catalytic applications. J. Am. Chem. Soc..

[81] Jiang Y., Liao J.-F., Xu Y.-F., Chen H.-Y., Wang X.-D., Kuang D.-B. (2019). Hierarchical CsPbBr_3_ nanocrystal-decorated ZnO nanowire/macroporous graphene hybrids for enhancing charge separation and photocatalytic CO_2_ reduction. J. Mater. Chem. A.

[82] Lin Z., Wang X. (2013). Nanostructure engineering and doping of conjugated carbon nitride semiconductors for hydrogen photosynthesis. Angew. Chemie.

[83] Ou M., Tu W., Yin S., Xing W., Wu S., Wang H., Wan S., Zhong Q., Xu R. (2018). Amino-assisted anchoring of CsPbBr_3_ perovskite quantum dots on porous g-C_3_N_4_ for enhanced photocatalytic CO_2_ reduction. Angew. Chemie.

[84] Guo X.-X., Tang S.-F., Mu Y.-F., Wu L.-Y., Dong G.-X., Zhang M. (2019). Engineering a CsPbBr_3_-based nanocomposite for efficient photocatalytic CO_2_ reduction: Improved charge separation concomitant with increased activity sites. RSC Adv..

[85] Kong Z.-C., Liao J.-F., Dong Y.-J., Xu Y.-F., Chen H.-Y., Kuang D.-B., Su C.-Y. (2018). Core@shell CsPbBr_3_@Zeolitic imidazolate framework nanocomposite for efficient photocatalytic CO_2_ reduction. ACS Energy Lett..

[86] Wan S., Ou M., Zhong Q., Wang X. (2019). Perovskite-type CsPbBr_3_ quantum dots/UiO-66 (NH_2_) nanojunction as efficient visible-light-driven photocatalyst for CO_2_ reduction. Chem. Eng. J..

[87] Hawecker J., Lehn J.-M., Ziessel R. (1983). Efficient photochemical reduction of CO_2_ to CO by visible light irradiation of systems containing Re(bipy)(CO)_3X_ or Ru(bipy)_3_^2+^–Co^2+^ combinations as homogeneous catalysts. J. Chem. Soc. Chem. Commun..

[88] Hawecker J., Lehn J., Ziessel R. (1986). Photochemical and electrochemical reduction of carbon dioxide to carbon monoxide mediated by (2, 2′-bipyridine) tricarbonylchlororhenium (I) and related complexes as homogeneous catalysts. Helv. Chim. Acta.

[89] Hori H., Johnson F.P.A., Koike K., Ishitani O., Ibusuki T. (1996). Efficient photocatalytic CO_2_ reduction using [Re(bpy)(CO)_3_{P(OEt)_3_}]^+^. J. Photochem. Photobiol. A Chem..

[90] Asai Y., Katsuragi H., Kita K., Tsubomura T., Yamazaki Y. (2020). Photocatalytic CO_2_ reduction using metal complexes in various ionic liquids. Dalt. Trans..

[91] Arias-Rotondo D.M., McCusker J.K. (2016). The photophysics of photoredox catalysis: A roadmap for catalyst design. Chem. Soc. Rev..

[92] Kong Z.-C., Zhang H.-H., Liao J.-F., Dong Y.-J., Jiang Y., Chen H.-Y., Kuang D.-B. (2020). Immobilizing Re(CO)_3_Br(dcbpy) Complex on CsPbBr_3_ Nanocrystal for Boosted Charge Separation and Photocatalytic CO_2_ Reduction. Sol. RRL.

[93] Chen Z., Hu Y., Wang J., Shen Q., Zhang Y., Ding C., Bai Y., Jiang G., Li Z., Gaponik N. (2020). Boosting Photocatalytic CO_2_ Reduction on CsPbBr_3_ Perovskite Nanocrystals by Immobilizing Metal Complexes. Chem. Mater..

[94] Que M., Zhao Y., Pan L., Yang Y., He Z., Yuan H., Chen J., Zhu G. (2020). Colloidal Formamidinium Lead Bromide Quantum Dots for Photocatalytic CO_2_ Reduction. Mater. Lett..

[95] Bresolin B.-M., Park Y., Bahnemann D.W. (2020). Recent progresses on metal halide perovskite-based material as potential photocatalyst. Catalysts.

[96] Li J., Cao H.-L., Jiao W.-B., Wang Q., Wei M., Cantone I., Lü J., Abate A. (2020). Biological impact of lead from halide perovskites reveals the risk of introducing a safe threshold. Nat. Commun..

[97] Flora G., Gupta D., Tiwari A. (2012). Toxicity of lead: A review with recent updates. Interdiscip. Toxicol..

[98] Chu L., Ahmad W., Liu W., Yang J., Zhang R., Sun Y., Yang J., Li X. (2019). Lead-free halide double perovskite materials: A new superstar toward green and stable optoelectronic applications. Nano-Micro Lett..

[99] Chakraborty S., Xie W., Mathews N., Sherburne M., Ahuja R., Asta M., Mhaisalkar S.G. (2017). Rational design: A high-throughput computational screening and experimental validation methodology for lead-free and emergent hybrid perovskites. ACS Energy Lett..

[100] Kamat P.V., Bisquert J., Buriak J. (2017). Lead-free perovskite solar cells. ACS Energy Lett..

[101] Fan Q., Biesold-McGee G.V., Ma J., Xu Q., Pan S., Peng J., Lin Z. (2020). Lead-Free Halide Perovskite Nanocrystals: Crystal Structures, Synthesis, Stabilities, and Optical Properties. Angew. Chemie Int. Ed..

[102] Volonakis G., Filip M.R., Haghighirad A.A., Sakai N., Wenger B., Snaith H.J., Giustino F. (2016). Lead-free halide double perovskites via heterovalent substitution of noble metals. J. Phys. Chem. Lett..

[103] Slavney A.H., Hu T., Lindenberg A.M., Karunadasa H.I. (2016). A bismuth-halide double perovskite with long carrier recombination lifetime for photovoltaic applications. J. Am. Chem. Soc..

[104] Zhou L., Xu Y., Chen B., Kuang D., Su C. (2018). Synthesis and Photocatalytic Application of Stable Lead-Free Cs_2_AgBiBr_6_ Perovskite Nanocrystals. Small.

[105] Wang Y., Huang H., Zhang Z., Wang C., Yang Y., Li Q., Xu D. (2020). Lead-Free Perovskite Cs_2_AgBiBr_6_@g-C3N_4_ Z-scheme System for Improving CH_4_ Production in Photocatalytic CO_2_ Reduction. Appl. Catal. B Environ..

[106] Wang X.-D., Huang Y.-H., Liao J.-F., Jiang Y., Zhou L., Zhang X.-Y., Chen H.-Y., Kuang D.-B. (2019). In situ construction of a Cs_2_SnI_6_ perovskite nanocrystal/SnS_2_ nanosheet heterojunction with boosted interfacial charge transfer. J. Am. Chem. Soc..

[107] Bhosale S.S., Kharade A.K., Jokar E., Fathi A., Chang S., Diau E.W.-G. (2019). Mechanism of Photocatalytic CO_2_ Reduction by Bismuth-Based Perovskite Nanocrystals at the Gas-Solid Interface. J. Am. Chem. Soc..

[108] Lu C., Itanze D.S., Aragon A.G., Ma X., Li H., Ucer K.B., Hewitt C., Carroll D.L., Williams R.T., Qiu Y. (2020). Synthesis of lead-free Cs_3_Sb_2_Br_9_ perovskite alternative nanocrystals with enhanced photocatalytic CO_2_ reduction activity. Nanoscale.

